# A randomized controlled cross-over trial of differences in acute effects on serum metabolites from isocaloric meals based on red meat, fatty fish, or soy protein

**DOI:** 10.1007/s00394-025-03710-0

**Published:** 2025-05-26

**Authors:** Helen M. Lindqvist, Erik Hulander, Linnea Bärebring, Inger Gjertsson, Anna Winkvist

**Affiliations:** 1 Department of Internal Medicine and Clinical Nutrition, Institute of Medicine, Sahlgrenska Academy, University of Gothenburg, PO Box 459, Gothenburg, SE-405 30 Sweden; 2 Department of Rheumatology and Inflammation Research, Institute of Medicine, Sahlgrenska Academy, University of Gothenburg, Gothenburg, Sweden; 3 Department of Rheumatology, Sahlgrenska University Hospital, Gothenburg, Sweden

**Keywords:** Rheumatoid arthritis, Metabolomics, Postprandial, Red meat, Fatty fish, Soy

## Abstract

**Purpose:**

Reducing red meat intake in the Western diet is beneficial for health and the environment. However, red meat is nutrient-rich, so understanding the impact of substituting it with other protein sources such as fish or plant-based proteins is essential, especially for vulnerable groups like the elderly and those with chronic diseases. The purpose of this study was to study the postprandial response in serum metabolites in women with Rheumatoid Arthritis (RA) after intake of red meat, fatty fish, and soy protein.

**Methods:**

Women with RA (*n* = 24) consumed isocaloric meals that included burgers made from either red meat, fatty fish, or soy protein in a crossover design. Blood samples were taken in fasting state before the meal (0 h) and at intervals up to 5 h after eating. Nuclear Magnetic Resonance (NMR) analysis quantified serum metabolites, and multivariate models and univariate statistics were applied to compare postprandial metabolite changes across protein sources.

**Results:**

Postprandial metabolite patterns varied significantly by protein type. The fatty fish meal led to a faster and higher increase in metabolites, including creatinine, isoleucine, valine, and trimethylamine N-oxide, compared to red meat. Unidentified lipids also differed. However, metabolite patterns after soy protein were similar to those after red meat.

**Conclusion:**

This postprandial crossover trial found that intake of fatty fish lead to a quicker and more pronounced increase in key blood concentrations of metabolites compared to red meat. However, metabolite profiles in serum based on NMR-analysis were similar after intake of soy protein compared to red meat.

**Trial registration:**

The PIRA (Postprandial Inflammation in Rheumatoid Arthritis) trial is Registered at Clinicaltrials.gov (NCT04247009).

**Supplementary Information:**

The online version contains supplementary material available at 10.1007/s00394-025-03710-0.

## Background

High intake of red meat is associated with increased risk of type 2 diabetes and cardiovascular disease in the general population in countries with high-sociodemographic index [1, 2]. Consequently, numerous studies have shown beneficial health effects of consuming fish or vegetarian alternatives instead of meat [3, 4]. In addition to negative effects on health, high meat consumption also has a negative impact on the climate [5]. Thus, current dietary recommendations in Sweden emphasize reducing intake of red meat and increasing intake of plant-based foods [6].

The recommendations of reduced meat consumption have spurred the development and availability of alternative products. A new generation of highly refined protein rich plant-based substitutes, designed to mimic the taste, texture, and presentation of meat, are providing means to moderate meat consumption with only minimal change in dietary habits. These products are not only consumed by vegans and vegetarians but by a larger part of the population [7] and are perceived to also have advantages in their environmental footprint, sustainability, and for animal welfare [8]. However, evidence of bioavailability, nutritional quality, and health benefits is still scarce. It is important to understand the consequences of substituting red meat for other protein sources, especially in vulnerable populations such as those at increased risk of poor nutritional status or low muscle mass. Patients with Rheumatoid Arthritis (RA) are at risk of low muscle mass [9, 10] and also report avoiding intake of red meat due to perceived negative effects on their disease [11, 12]. Thus, patients with RA may be a vulnerable population within the context of the protein shift.

Animal protein sources such as beef, pork, poultry, fish and dairy have high protein quality, i.e. high digestibility and include sufficient amounts of all essential amino acids such as valine, isoleucine, leucine, methionine, phenylalanine, tryptophan, threonine, histidine, and lysine [13]. In addition, research increasingly suggests that dietary protein quality depends not only on amino acid composition and digestibility but also on the actual metabolic availability rate of amino acids. A higher rate has been reported to be beneficial for supporting rapid muscle mass and functional recovery in conditions such as critical illness or chronic diseases that lead to loss of muscle mass [14]. So far, most trials studying protein quality including the postprandial aspect of “fast” or “slow” proteins have focused on dairy products such as whey and casein proteins [14, 15]. There are also a number of studies of different protein sources such as meat [16, 17], fish, or plant-based products [16, 18–22] that report postprandial amino acid concentrations in plasma or serum at several time points, but without focus on rate of increase. Thus, knowledge of differences in postprandial kinetics of amino acid absorption from different protein sources is still limited, but of great importance in this time of protein shift.

In sum, there are numerous advantages in exchanging red meat for fish or plant-based protein, but knowledge on how different protein sources influence the concentration of amino acids and other metabolites in serum postprandially is limited. The aim is therefore to determine how different dietary protein sources affect postprandial metabolites in serum, especially amino acids, in patients with RA.

## Methods

The randomized crossover trial PIRA (Postprandial Inflammation in Rheumatoid Arthritis) was approved by the Swedish Ethical Review Authority (Dnr 2019–05242) and registered at Clinicaltrials.gov (NCT04247009). The study was initiated in February of 2020 and the last meals were served in November 2021.

### Recruitment

Women with diagnosis of RA since at least two years, aged 20–70 years and living in the area around Gothenburg were identified through the Swedish Rheumatology Quality Register. An additional inclusion criterion was body mass index (BMI) 18.5–30.0 kg/m^2^. Exclusion criteria were smoking, allergy or intolerance to any of the study foods or unwillingness to consume the study meals, pregnancy or breastfeeding, diagnosis of cancer, diabetes, inflammatory bowel disease or celiac disease, use of lipid lowering medication, glucocorticoids or interleukin-6 (IL-6) inhibiting therapy during the past 4 weeks, hemoglobin levels < 100 g/L or glycated hemoglobin (HbA1c) above reference range. The 26 allocated study participants have been described in detail previously [23].

### Screening

At screening, data on height and weight were collected. C-reactive protein (CRP), erythrocyte sedimentation rate (ESR), hemoglobin and HbA1c were measured in fresh blood samples by the laboratory at the Sahlgrenska University Hospital, Gothenburg, Sweden. Disease Activity Score 28-joints (DAS28) using ESR was estimated by nurses at the Department of Clinical Rheumatology research center at the Sahlgrenska University Hospital. Information on the participants’ medications was collected from patient records and confirmed during interviews. Participants also filled out a lifestyle questionnaire previously described in detail [24] and answered a Food Frequency Questionnaire (FFQ) reflecting the past 12 months, with questions about 53 food items. Physical activity was assessed based on scales between 1 and 5 on habitual physical activity and intentional physical exercise. Based on this, a physical activity index between 1 and 4 was calculated, resembling that previously validated by Wareham et al. [25].

Due to the Covid-19 pandemic, the PIRA trial was performed in two periods, one in January to March 2020 and one between August 2021 to November 2021.

### Intervention

Meals were ingested in the morning after an overnight fast. Participants were told to avoid supplementation, if not prescribed by physician, during the study period (from inclusion until the last study meal day was finished) and any exercise the evening and morning before test meals. Participants meals were rescheduled if they had an ongoing temporary infection, such as a cold. The meal sequence was randomized by computer generated randomization (Microsoft Excel using the RAND()-function). The sequence allocation was done post screening, so that the staff did not know the meal order at screening. A glass of water was served with every meal, and participants consumed the meal within 20 min. They were also allowed to drink water freely throughout the day.

### Study meals

The intervention meals were served in the form of hamburgers and have been described in detail previously [23]. In short, the burger recipes were designed to ensure comparable distribution of energy derived from protein, carbohydrates, and fat across all three meals, as delineated in Table 1. The burgers were made from either minced meat (60% beef, 40% pork, produced by Scan, from Swedish meat products), minced fresh salmon (Farmed by Salmar Farming AS, Norway, grinded by Kullaviks fisk, Sweden) or a soy protein mince (soy based vegan substitute with product name Anamma Formbar Färs, produced by Orkla Foods, Sweden. Ingredients; water, soy protein (23%), canola oil, salt, spices, natural aromas, caramelized sugar, stabilizing agent (methylcellulose)).

To maintain uniformity in the cooking process, all burgers were fried in non-stick pans with 1 teaspoon of canola oil. Specific temperature thresholds were applied during the cooking procedure: minced meat burgers were cooked until the internal temperature reached 75 °C, fish burgers to 52 °C, while soy protein burgers were heated to an internal temperature of 70 °C. The burgers were thereafter frozen and stored at a temperature of -18 °C for maximum 3 months prior to consumption. Samples of the cooked burgers underwent macronutrient analysis externally at Eurofins Food & Feed Testing in Sweden. This analysis was followed by adjustments to the dressing fat content, involving the addition of canola oil as required to standardize the energy and macronutrient profiles of each meal, as delineated in Table 1. The soy protein burger provided ~ 10 g dietary fiber from the soy protein mince, which was not adjusted for.

The side items were the same for all meals, and comprised 2 slices of toast, ~ 10 g romaine lettuce, ~ 10–20 g cucumber, ~ 20–30 g tomato, and a vegan dressing. Due to the study being halted because of the pandemic, the burgers were cooked in two batches.

**Table 1:** Nutritional content of the burgers and served meals^1^

	Red meat^2^	Fatty fish^3^	Soy protein^4^
**Burgers**			
Weight (g)	141	146	177
Energy (kcal)	337	327	301
Protein^5^ (g)	27.1	26.6	31.3
Carbohydrate^6^ (g)	5.9	5.9	5.6
Fat^5^ (g)	22.9	22.0	17.1
**Meal total**			
Energy (kcal)	692	682	709
Protein (g)	34.7	34.2	38.8
Carbohydrate (g)	46.8	46.8	46.4
Fat (g)	40.8	39.9	41.0

^1^Table illustrates the batch produced in 2021

^2^Burger from red meat (60% beef and 40% pork) produced by Scan, by Swedish meat products

^3^Burger from fatty fish (Salmon) farmed by Salmar Farming AS, Norway

^4^Burger from soy based vegan substitute with product name *Anamma Formbar Färs*, produced by Orkla Foods, Sweden

^5^Data analyzed by Eurofins Food & Feed Testing, Sweden

^6^Data calculated based on food labels, fiber content not included

### Outcomes

The main outcome in the current report was differences in changes in postprandial metabolite patterns at 3 h after intake of the study meals, between red meat versus fatty fish and soy protein meals. Secondary outcomes were differences in metabolite patterns and concentrations at other postprandial timepoints, as well as differences in incremental area under the curve based on the minimum value (AUC_min_) for quantified metabolites. The main outcome in the PIRA trial was IL-6, which has been reported previously [23].

### Blood sampling

During the postprandial meal challenges, a venous catheter was inserted to facilitate blood collection. Blood samples were obtained in the fasting state (0 h) and at 30 min, one hour, two hours, three hours, and five hours following consumption of the meals. Serum was separated by allowing blood samples to rest in BD Vacutainer tubes (5 mL, reference no 367624) at room temperature for 30 min, followed by refrigeration for an additional 30 min, before undergoing centrifugation at 2600 g for 10 min. Subsequently, all collected serum samples were promptly preserved at -20 °C and, at the earliest opportunity (within a couple of hours), transferred to long-term storage at -80 °C until the time of analysis.

### Analysis method

#### Nuclear magnetic resonance (NMR)-analysis

Serum samples were prepared according to IVDr standard operating procedures (Bruker BioSpin; www.bruker.com/products/mr/nmr/avanceivdr.html). Serum samples were thawed at room temperature for 30 min, centrifuged at 3500 x g for 1 min at 4 °C and transferred (325 µl) with a SamplePro L liquid handler (Bruker BioSpin) to a deepwell plate (Porvair, cat. No 53.219030). The wells were prepared with 325 µl NMR buffer (75 mM sodium phosphate, pH 7.4, 0.08% 3-(trimethylsilyl)propionic-2,2,3,3- d4, 0.04% sodium azide, 20% v/v D_2_O) and the plate was shaken at 400 rotations per minute, 12 °C for 5 min in a Thermomixer Comfort (Eppendorf). Finally, 600 µl sample was transferred to 5 mm SampleJet NMR tubes with the SamplePro L. The sample tubes, deepwell plate and SampleJet rack were kept at 2 °C during the preparation in the SamplePro L robot. A pre-acquisition temperature stabilization time of 300 s was used and ^1^H NMR data wereas acquired on a Bruker 600 MHz Avance III spectrometer equipped with a room temperature 5 mm BBI probe and a cooled SampleJet sample changer where sample racks were kept at 6 °C before data acquisition. The 1D NOESY (‘noesygppr1d’ pulse sequence), 1D CPMG (‘cpmgpr1d’) and 2D J-resolved (‘jresgpprqf’) spectra were acquired according to the standard IVDr parameter settings at 37 °C. Experimental parameters are available upon request.

For Bruker IVDr quantification protocol 1D NOESY data were submitted for automatic quantification of a subset of metabolites through a remote secure Bruker server, generating in total 112 B.I.Lisa and 41 B.I.Quant-PS variables. The procedure followed the IVDr quantification protocol that can identify and quantify 39 metabolites (mainly amino acids, carboxyl acids, keto acids, and a few sugars).

The spectra were also bucketed for multivariate modelling of serum metabolite patterns. The ^1^H-NMR spectra were then referenced to the glucose anomeric proton doublet at 5.23 ppm and the function “opt_bucket.m” [26] was used with the size of bucket = 0.02 ppm and slackness = 0.5.

### Data curation and preparation for quantified metabolites

For the metabolites quantified by Bruker IVDr Quantification in Plasma/Serum B.I.Quant-PSTM we used the ρ-value to exclude data with low quality. The ρ -value describes the correlation of line shape metabolite signal with calculated fit characterizing the match between metabolite signal and fit. Metabolites where more than half the samples had less than 85% correlation were excluded from all analysis. The remaining 23 included quantified metabolites were: acetone, creatine, creatinine, N,N-dimethylglycine, trimethylamine-N-oxide (TMAO), glucose, dimethylsulfone, the amino acids alanine, glutamine, glycine, tyrosine, and the essential amino acids histidine, isoleucine, leucine, phenylalanine, valine, the acids acetic acid, citric acid, formic acid, lactic acid, succinic acid, acetoacetic acid, and pyruvic acid. For these, AUC_min_ was calculated by subtracting the lowest concentration from all values for each individual before AUC was calculated following the method for incremental AUC_min_ by Brouns et al. [27]. For the bucket data, AUC_min_ was calculated for all individual buckets (*n* = 284) between the chemical shift range of -0.1–6.0 ppm, excluding the water peak at 4.4–4.8 ppm.

### Statistical analysis

#### Univariate methods

Friedman test was used to compare AUC_min_ for the three meals for all quantified metabolites. If the p-value was less than 0.05 for AUC_min_, Friedman test was also used to compare that quantified metabolite at each time point. Wilcoxon signed rank test was used for post-hoc analysis for pairwise comparisons of quantified metabolites and for selected buckets from the metabolomics data set. To adjust for multiple testing, a Bonferroni correction was applied; the 284 buckets represent ∼70 metabolites and with a margin we adjusted for 100 tests, i.e., P values < 0.0005 were regarded as significant. Univariate analyses were carried out using SPSS version 29.0.

#### Multivariate methods

Multivariate data analysis using principal component analysis (PCA), orthogonal projections to latent structures with discriminant analysis (OPLS-DA) [15], and orthogonal projections to latent structures with effect projection (OPLS-EP) [16] were performed by SIMCA software v.18 (Umetrics AB, Umeå, Sweden). Prior to modeling, all data were scaled to unit variance or unit variance none for OPLS-EP. A PCA model was used to explore clustering patterns of observations, trends in the data, and outliers for the fasting samples (0 h) from the first meal served. OPLS-DA was used to compare changes between the three meals for AUC_min_ and at 3 h. In addition, OPLS-EP models, developed for paired samples, were used to identify discriminating metabolites that changed within 3 h after each meal and to compare AUC_min_ for the three meals. The validity of the models was assessed by cross validation, permutation tests (*n* = 999), Coefficient of Variation-Analysis Of Variance testing of Cross-Validated predictive residuals (CV-ANOVA) and the cumulative amount of explained variation in the data summarized by the model (R2X[cum] and R2Y[cum]).

To select class-discriminating buckets of interest for annotation, the 20th top-ranked buckets in variable importance scores (VIP) in the OPLS models with loadings (w ≥ ± 0.1) were assessed. Discriminating buckets were only selected and identified if the model had a CV-ANOVA p-value less than 0.05.

### Power calculation

The power calculation for the PIRA trial was based on the study main outcome IL-6 [23]. This calculation is not applicable for the metabolomics outcomes. There are few studies on postprandial metabolite patterns, but significant differences have been found in our previous three meal cross-over interventions with a sample size of about 20 participants/group [28–30], indicating that this is a sufficient group size.

## Results

**Fig. 1:**
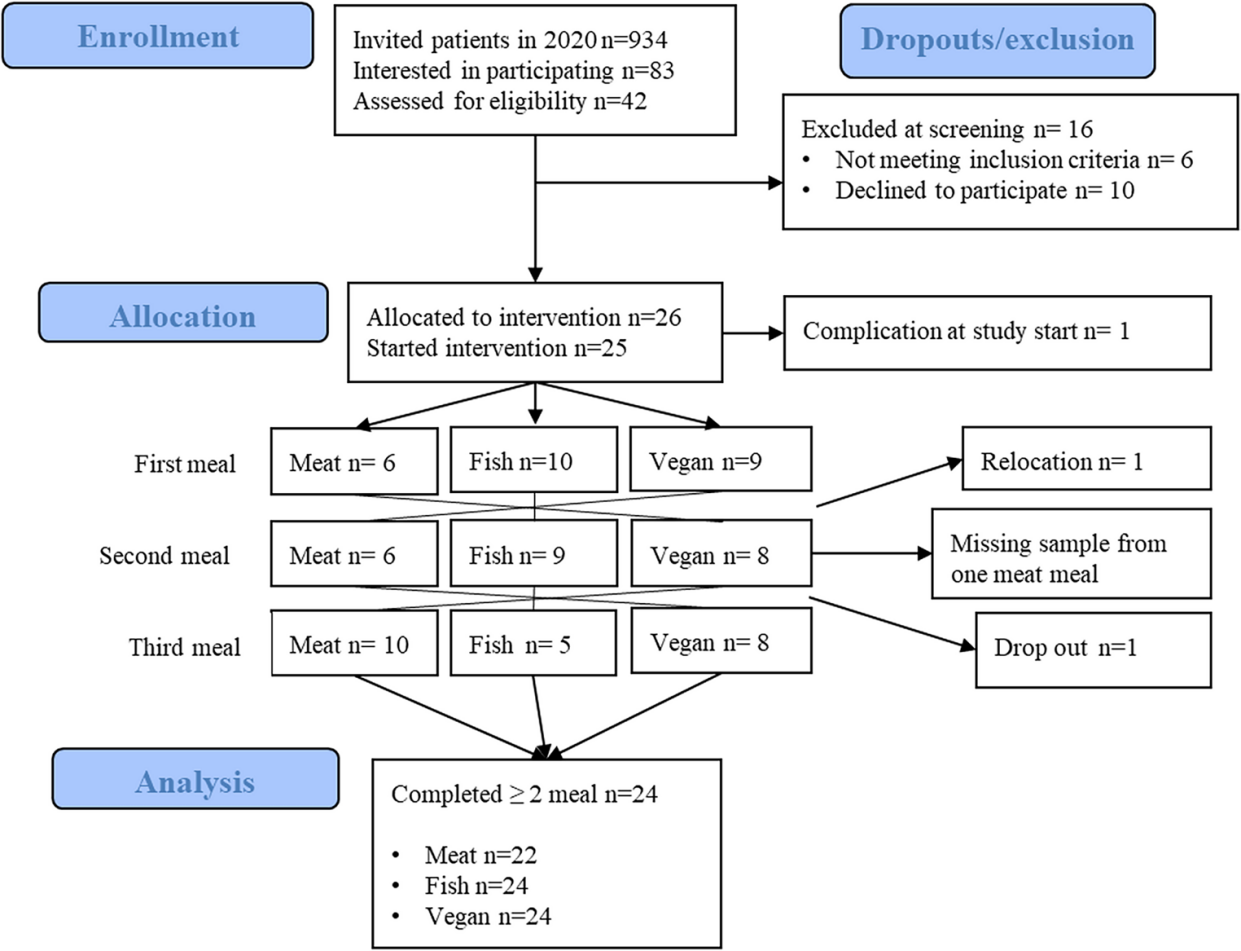
Flow chart of PIRA trial reported according to CONSORT

In total, 934 women diagnosed with RA were contacted by letter. Eighty-three people responded, and 43 of those fulfilled the inclusion criteria and were invited to a screening visit. Subsequently, 26 were assigned to a meal sequence (Fig. 1**)**. In total, 24 completed at least two meal challenges, and 22 participants completed all three meal challenges.

All participants had a European origin, and the majority were in remission according to DAS28, had a high educational level, and the age span was between 46 and 71 years when consuming the meals. (Table 2). All but three participants had passed menopause and only one individual used hormonal contraceptives.

**Table 2:** Baseline demographic characteristics of participants completing at least 2 meal challenges in PIRA trial (*n* = 24)

	Median (p25, p75)
Age (year)	66 (60, 69)
Waist (cm)	85 (77, 91)
Hip (cm)	102 (97, 108)
Body mass index (kg/m^2^)	24.6 (23.2, 26.8)
Erythrocyte sedimentation rate (mm/1 h)	11 (6, 22)
C-reactive protein (mg/L)	1.3 (0.6, 3.0)
DAS28	2.5 (1.9, 3.4)
Global Health^1^ (0–100 mm)	14 (3, 32)
Tender joints (no)	1.0 (0.0, 4.5)
Swollen joints (no)	0.0 (0.0, 1.8)
Triglycerides (mg/dL)	73 (64, 93)
Physical activity^2^	**n (%)**
Inactive	3 (13)
Moderately inactive	5 (21)
Moderately active	4 (17)
Active	12 (50)
Educational level	
Junior high school	1 (4)
2 years senior high school	3 (13)
≥ 3 years senior high school	1 (4)
University	19 (79)
Working status	
Not working	10 (42)
Working fulltime (≥ 40 h a week)	4 (17)
Medication	
Biologic DMARD	10 (42)
Conventional synthetic DMARD	22 (92)

^1^Self-assessed on a visual analog scale, ranging from 0 to 100 mm

^2^Based on scales between 1 and 5 on both habitual physical activity and intentional physical exercise, an activity index between 1 and 4 was calculated, resembling what was previously tried and validated by Wareham et al. [25], commonly referred to as the Cambridge Index. DAS28, 28-joints disease activity score erythrocyte sedimentation rate; DMARD, Disease modifying anti-rheumatic drugs

### Results multivariate modelling of changes over time of bucketed NMR-spectra

**Fig. 2:**
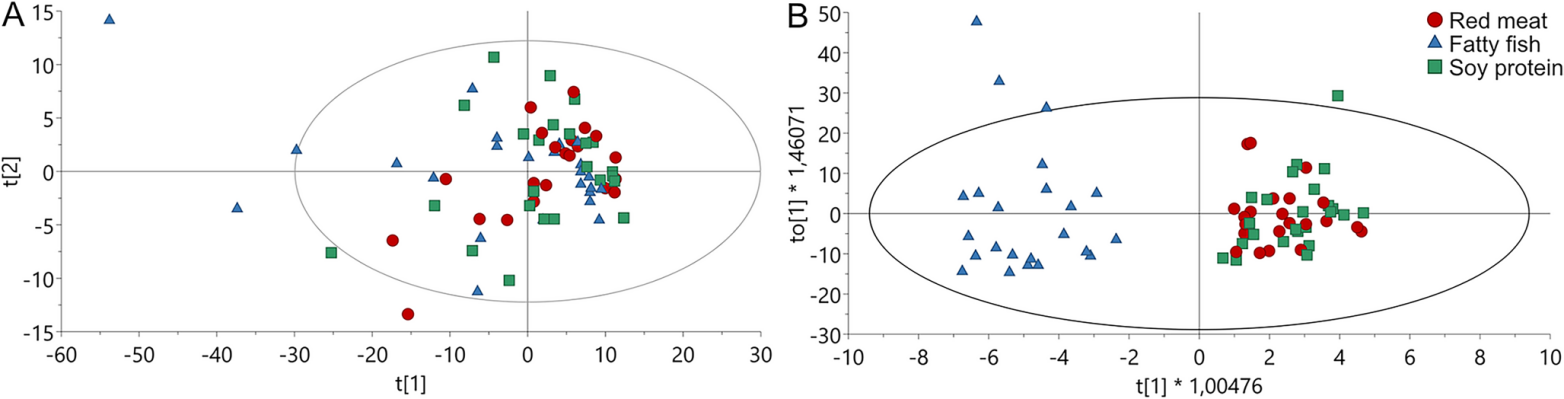
PCA-X (**A**) and OPLS-DA (**B**) with AUC_min_ based on “buckets” for red meat, fatty fish, and soy protein meals. Red circles = red meat, blue triangle = fatty fish, green box = soy protein

Postprandial models comparing 3 h samples to 0 h (fasting) samples in OPLS-EP models showed significant changes for all three meals (Table 3, model 4, 5, 6). The PCA model for AUC_min_ for buckets did not separate between the three meals (Table 3, model 2, Fig. 2A). But, comparing the AUC_min_ for buckets for all three meals in an OPLS-DA model (Table 3, model 3) resulted in a high quality model and showed sigificant differens in metabolite patterns for fatty fish compared to the other meals in the OPLS-model (Fig. 2B). Comparing red meat and fatty fish on either AUC_min_ or at 3 h postprandial, using OPLS-DA (Table 3, model 7, 9) and OPLS-EP models (Table 3, model 8), resulted in high quality models and good predictability. Comparing red meat and soy protein meals with OPLS-DA (samples *n* = 46) or OPLS-EP (*n* = 22) did not result in any models at all.

Selected buckets (criteria: VIP top 20 and loadings w ≥ ± 0.1) from OPLS-EP and OPLS-DA models are presented in supplemental Table 1. For red meat, no buckets fullfilled the selection criteria when comparing 0 h (fasting) and 3 h samples (model 4, Table 3). For fatty fish, creatinine was the only metabolite that fullfilled the selection criteria comparing 0 h (fasting) and 3 h samples (model 5, Table 3). For the soy protein meal, however, several unidentified lipids and buckets including a mix of metabolites increased significantly over time and fullfilled the selection criteria (model 6, Table 3). In the models comparing changes in buckets between 0 h (fasting) and 3 h and AUC_min_ between red meat and fatty fish, buckets representing creatine, isoleucine, leucine, and some lipids fullfilled the selection criteria and where higher in concentration for fatty fish. This was in line with the findings from the quantified serum metabolites.

**Table 3:** Models statistics for NMR-data in buckets

Model		Nr of Lv^1^	*N*	R2X [cum]^2^	R2Y [cum]^3^	Q2 [cum]^4^	CV-ANOVA^5^ (*p*-value)	ROC AUC^6^	Class % (red meat/fatty fish/soy protein)	Permutation test (Q2)^7^
**1**	PCA-X All meals first visit T0	3	24	0.599		0.300				
**2**	PCA-X All meals AUC_min_	5	70	0.707		0.605				
**3**	OPLS-DA All meals AUC_min_	1 + 3 + 0	70	0.606	0.461	0.310	0.0000004	0.71/1.0/0.82	23/100/83	-0.223
**4**	OPLS-EP Red meat 0 h vs. 3 h	0 + 0 + 0	22							
**5**	OPLS-EP Fatty fish oh vs. 3 h	1 + 2 + 0	24	0.751	0.938	0.872				
**6**	OPLS-EP Soy protein 0 h vs. 3 h	1 + 2 + 0	24	0.653	0.945	0.630				
**7**	OPLS-DA Red meat vs. Fatty fish Δ3h	1 + 4 + 0	46	0.672	0.945	0.630	0.0000330	1.0/1.0	100/100	-0.564
**8**	OPLS-EP Red meat vs. Fatty fish AUC_min_	1 + 3 + 0	22	0.730	0.985	0.725	0.0063470			
**9**	OPLS-DA Red meat vs. Fatty fish AUC_min_	1 + 4 + 0	46	0.703	0.960	0.565	0.0003772	1.0/1.0	100/100	-0.525

NMR = Nuclear Magnetic Resonance, PCA = principal component analysis, OPLS-EP = orthogonal projections to latent structures with effect projection, OPLS-DA = orthogonal projections to latent structures with discriminant analysis, AUC_min_=Area under curve minimum. 1 Latent Variables, 2 Cumulative fraction of the sum of squares of X explained by the selected latent variables, 3 Cumulative fraction of the sum of squares of Y explained by the selected latent variables, 4 Cumulative fraction of the sum of squares of Y predicted by the selected latent variables, estimated by cross validation, 5 ANalysis Of VAriance testing of Cross-Validated predictive residuals, 6 ROC AUC = Receiver Operating Curve Area under curve, 7 The intercept between real and random models, degree of overfit

### Postprandial effects on quantified serum metabolites

**Fig. 3:**
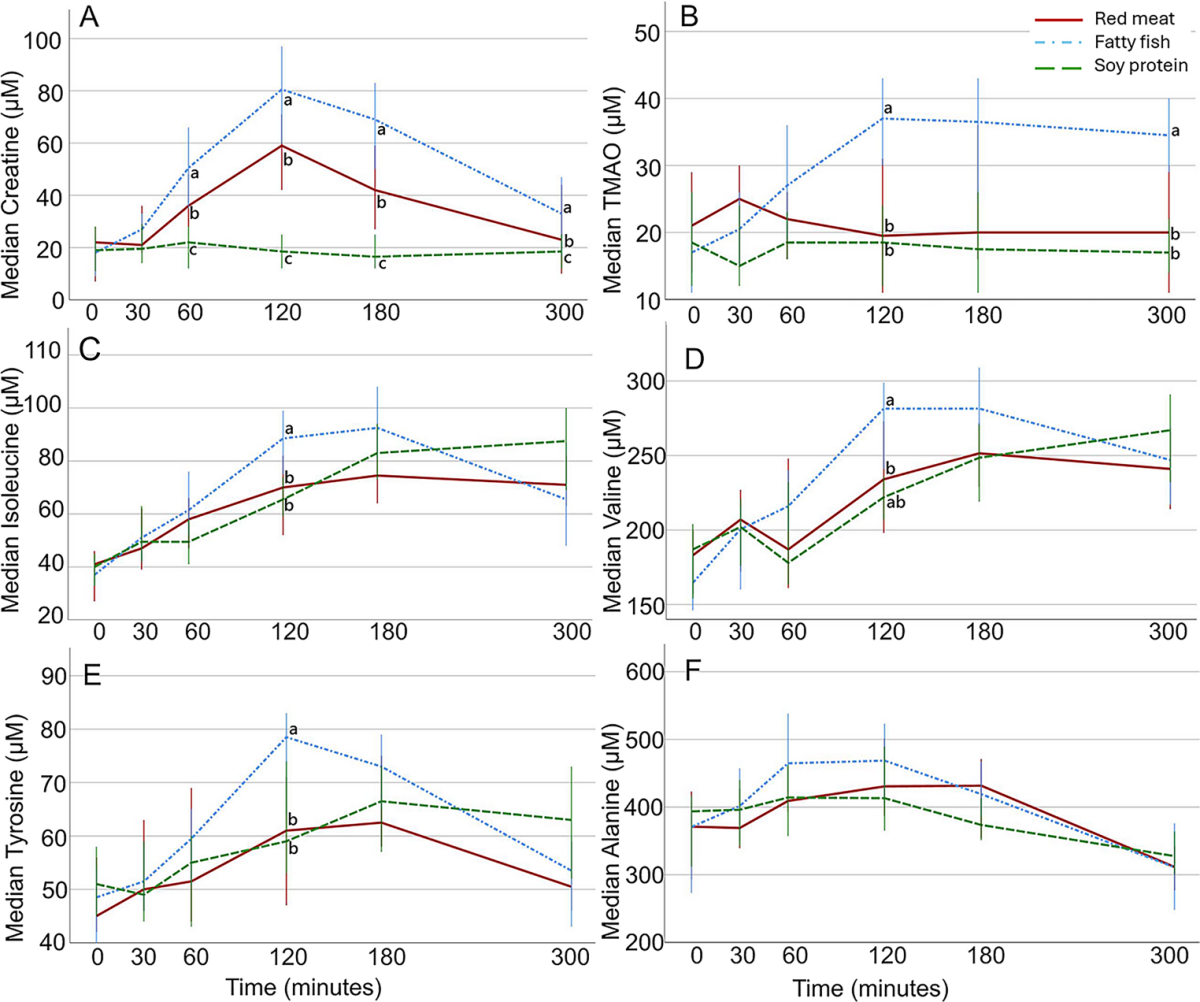
Median serum concentrations (mmol/l) 0 h (fasting) and post meal intake for (**A**) creatine (**B**) trimethylamine N-oxide (TMAO) (**C**) isoleucine (**D**) valine (**E**) tyrosine (**F**) alanine Different letters (a, b, c) indicate significant different concentrations at that time point (*p* < 0.0005) for Wilcoxon signed rank test. Solid red line = red meat, dotted blue line = fatty fish, dashed green line = soy protein

Serum metabolites did not differ significantly in fasting samples (0 h). Seven out of 23 metabolites had differences (Friedman test *p* < 0.05) in AUC_min_ between the three meals (Table 4). Metabolites with either a significant difference at any time point or with a significant AUC_min_ (*p* < 0.0005) are presented in Fig. 3. Of these, creatine, TMAO and alanine had a significantly (*p* < 0.0005) different AUC_min_ between the three meals. Creatine had the greatest difference between the meals and significant differences at one, two, three and five hours with no increases after the soy protein meal and the highest increase after the fatty fish meal (Fig. 3A). TMAO only increased after the intake of the fatty fish meal (Fig. 3B), with the largest differences between fatty fish and the other two meals at 2 h and 5 h (*p* < 0.0005). Tyrosine (Fig. 3E), isoleucine (Fig. 3C) and valine (Fig. 3D) increased faster and to a higher concentration after intake of the fatty fish meal compared to after intake of red meat and soy protein meals, with a significant difference at 2 h postprandial. The trend was similar for alanine (Fig. 3F), but the difference was not significant.

**Table 4:** IVDR quantified metabolites with a *p*-value < 0.05 for AUC_min_ comparing meals

	AUC_min_^1^	Significance at different time points^1^						Post hoc analysis^2^			
Metabolite	*p*	*p* 0 min	*p* 30 min	*p* 1 h	*p* 2 h	*p* 3 h	*p* 5 h	1 h	2 h	3 h	5 h
Creatine	7.2 × 10^− 10^	0.657	0.228	4.1 × 10^− 7^	1.1 × 10^− 8^	7.2 × 10^− 10^	6.3 × 10^− 5^	All^3^	All^3^	All^3^	All^3^
TMAO	2.3 × 10^− 6^	0.645	0.054	0.104	2.5 × 10^− 4^	9.0 × 10^− 3^	2.1 × 10^− 5^		Fatty fish^4^		Fatty fish^4^
Alanine	4.6 × 10^− 4^	0.438	0.580	0.018	0.014	0.021	0.639				
Puruvic acid	1.8 × 10^− 3^	0.957	0.183	0.344	0.966	0.043	0.094				
Tyrosine	3.6 × 10^− 3^	0.497	0.812	0.008	1.3 × 10^− 5^	0.081	0.015		Fatty fish^4^		
Isoleucine	7.4 × 10^− 3^	0.743	0.709	0.002	4.1 × 10^− 5^	0.075	6.1 × 10^− 4^		Fatty fish^4^		
Valine	2.2 × 10^− 2^	0.420	0.048	0.012	3.9 × 10^− 5^	0.002	0.302		Fatty fish differ to red meat^5^		

(1) Friedman test between all three meals at each time point (2) Pairwise testing with Wilcoxon signed rank test (3) All (red meat, fatty fish and soy protein) significantly different from each other (4) Fatty fish significantly different compared to red meat and soy protein (5) Fatty fish significantly different compared to red meat, AUC_min_=Area Under Curve minimum

## Discussion

This study found that red meat, fatty fish, and soy protein resulted in differential metabolite patterns and differences in both timing and total AUC_min_ for quantified metabolites in serum postprandial samples. The fatty fish meal resulted in a faster increase and higher concentrations of several protein metabolites compared to red meat and soy protein meals. It is important to point out that this study was not designed to study the bioavailability of amino acids, but the changes in metabolites patterns in serum postprandially. However, our data included twelve quantified amino acids and derivates of these. In addition, the metabolite patterns indicated that also lipid metabolites in serum discriminated between the protein sources, but additional analysis of specific lipid species is necessary to characterize these differences properly.

Our results show several expected findings such as the increase in creatine, a muscle protein, after the fatty fish and red meat meals, but not after the soy protein meal. Similar results have been reported comparing postprandial effects on specific metabolites, including amino acids, from beef, pork, and chicken and a vegetarian product (green pea and egg white) [18]. According to a recent review on food metabolites, creatine was considered a fair marker for intake of meat and a good marker for fish [31]. However, beef and salmon have the same content of creatine (4.5 g/kg) [32], still our result showed that creatine increased faster and to a higher concentration after fish consumption, compared to beef. We speculate that the lower level of collagen in fish compared to beef (0.28% versus 0.59%) [33], could result in a faster digestion of the muscle meat and explain this result. An in vitro digestibility trial reported that the digestibility of fish is greater than that of beef (47% versus 43%) and that the peptides found after in vitro treatment of beef and fish differed independent of in vitro treatment (pepsin or pepsin and trypsin) or in vivo assay (with gastric content or with jejunal contents) [33]. Also as expected, TMAO increased significantly after the fatty fish meal, but not after the red meat and soy protein meals. Fish and shellfish contain TMAO, and TMAO has been found to increase significantly in postprandial studies of fish previously [21]. TMAO has also been evaluated to be a good candidate marker for intake of fatty fish [31].

Moreover, a consistent pattern emerged with respect to the amino acids valine, isoleucine, and tyrosine, indicating a swifter and more pronounced elevation following the consumption of fatty fish when compared to the other two meals. This finding do not align with findings from a similar study by Schmedes et al. [21], that found no postprandial difference in valine and isoleucine. On the other hand, Schmedes et al. compared meals that included pasta, sauce, vegetables, a cinnamon bun, and 20 E% of cod or lean beef [20] whereas the burgers (red meat, fatty fish and soy protein) in our meals constituted 42–49 E%. Hence, the meals differed both in the energy percentage of protein source and type of fish and meat [21]. These differences might explain the divergent findings.

Given the matched total protein content in the PIRA meals, one might anticipate a slightly diminished presence of valine, isoleucine, and alanine in serum after the soy protein meal relative to the red meat and fatty fish meals, in accordance with the amino acid composition in these sources. However, this was not the case, further indicating that the amino acid composition of the food is not the only factor influencing amino acid concentrations in serum postprandial. Due to anti-nutritional factors including trypsin inhibitors, protease, tannins, and phytates present in soybean, one could anticipate a lower uptake of amino acids from soy products, but our results indicate the contrary. Production process can reduce anti-nutritional factors, which could be the case with the soy mince served [34]. In addition, the postprandial increase of valine, isoleucine, and tyrosine was followed by a slower decline in the levels of these amino acids after the soy protein meal in comparison to the animal-based meals. This could indicate that the food matrix, even though all meals consisted of cooked burgers of grinded food, has a significant impact on the amino acid release in the digestion system. Gastric emptying is dependent on how easy the food can be fragmented, and this could be influenced by the content of anti-nutrients delaying protein degradation and other food matrix structures [35]. Differences in postprandial amino acids have been shown after different dairy matrixes [15], after mycoprotein/pea protein in the form of beverage or high-moisture extrusion [17], and after meat from chicken and pork [16]. In a similar setting to ours, but with slightly smaller meals (sauce instead of the bread to the burgers), Ottosson et al. [22] observed no postprandial differences in concentrations of isoleucine and tyrosine after intake of dairy, fish (salmon and cod), meat or legumes. The concentrations of these amino acids also dropped much faster than in our study. However, compared to PIRA, the power of their study was lower due to fewer participants in each group, slightly lower protein content and younger and healthier participants, which could explain the dissimilarities between our findings. Pham et al. [19] compared different types of meat (pasture fed beef, grain fed beef, and lamb) to a pea-based meat substitute. They concluded that AUC for serum concentrations for many amino acids, including isoleucine, valine, and tyrosine, differed between these meals, indicating that even meat of the same animal origin might provide different amounts of amino acids. However, although the pea-based product had a similar content of isoleucine, valine, and tyrosine as the other meals in their study, the AUC concentration in plasma was much lower than from the animal-based meals, indicating differences in uptake and/or metabolism. This was not the case in our study where the soy protein product resulted in similar serum concentrations as after red meat. In fact, plant foods have different composition and type of dietary fiber and the production methods and steps for meat analogues differ vastly. Thus, it can be anticipated that the bioavailability of different nutrients varies between these products even when produced from the same raw material. This emphasizes the importance of evaluating the nutrient bioavailability in new vegan protein products, when replacing meat.

### Postprandial metabolites and possible effects on health

Postprandial metabolites, except glucose, have few direct established associations with health outcomes. For example, the postprandial TMAO, reported here, has likely nothing to do with the steady-state TMAO concentrations measured in fasting state. The TMAO concentrations in fasting state are presumed to be affected by gut microbiota activity and meat consumption, and it has been reported in a meta-analysis that elevated plasma TMAO concentrations increase the risk of all-cause mortality [36]. A more recent study reported that TMAO directly correlates with blood pressure and BMI in a population with relatively low fish intake, but not in a population with relatively high fish intake [37]. It is likely that TMAO concentrations depend on the overall background diet of a population, and that TMAO does not represent an independent CVD risk factor. Finally, it has been suggested that during aging, protein gain is greater with rapidly digested protein than with slowly digested protein [38]. If the digestion rate is of importance for muscle mass, the finding that fatty fish burgers made of salmon gave a swift increase of amino acids in postprandial serum could be of interest to further investigate in older adults with sarcopenia or in patients with cachexia or other muscle degenerative conditions.

### Strengths

We have devised an isocaloric randomized cross-over intervention with a standardized macronutrient profile, thus enabling us to interpret the results without the confounding of energy content or macronutrient composition, as has frequently been the case in prior investigations. The selection of a meal size ranging from 600 to 700 kilocalories, along with the composition of macronutrients, mirrors a realistic meal composition. Consequently, this design permits us to formulate hypotheses concerning the potential impacts of food choices in the context of real-world dietary behaviors. Further, the structural characteristics of food have been observed to affect the absorption of amino acids, as demonstrated by Pennings [39]. Therefore, an important strength of our study lies in the comparative analysis of minced red meat, minced fatty fish, and a corresponding soft soy-based product. This reduces the confounding factor of the food structure, which could influence digestion, uptake, and time for metabolites to reach the blood.

### Limitations

To maintain consistency in fat content across meals, a minor amount of canola oil (6 g) was added to the soy protein meal. Though the quantity of added oil was small, it accounted for approximately 15% of the total fat in the soy protein meal, potentially exerting a minor impact on the outcomes. However, red meat (pork and beef) and fatty fish (salmon) shared similar macronutrient compositions and required no adjustments. NMR-analysis results in limited information on lipid species and additional methods of analysis would add more information about the effects of the intervention on these.

The generalizability of our findings may be limited since our sample predominantly comprised middle-aged women with RA. However, we have recently published data from the same trial showing that few metabolites differed after ingestion of the meal with red meat between the women with RA and a matched control group without RA [40]. In fact, none of the metabolites that differed between the dietary protein sources in the current report differed between women with and without RA after the meat meal, indicating that our results may be relevant also to those without RA.

## Conclusion

In conclusion, our study revealed significant variations in postprandial serum metabolite patterns and quantified metabolites based on the protein source in the ingested meals in women with RA. Notably, postprandial creatine and TMAO levels reflected the content of these in muscle meat (meat and fish) and fatty fish, respectively. We observed a consistent pattern of faster and more pronounced increases in valine, isoleucine, and tyrosine after fatty fish consumption compared to after the other meals, along with a slower decline after the soy protein meal. The vegan soy protein product did not result in a lower serum concentration of amino acids than did minced red meat, indicating that this is a rather good substitute for meat from a protein point of view. However, when substituting meat with vegan products intake of other important nutrients, such as minerals, should be considered.

Further studies are needed to fully understand the implications of dietary choices on postprandial metabolic responses and the subsequent health impact across diverse populations.

## Electronic supplementary material

Below is the link to the electronic supplementary material.

Supplementary Material 1
