# Supplementary material for: A randomized controlled cross-over trial of differences in acute effects on serum metabolites from isocaloric meals based on red meat, fatty fish, or soy protein
Source: Eur J Nutr. 2025 May 26;64(5):187. doi: 10.1007/s00394-025-03710-0 (PMC12106552; doi:10.1007/s00394-025-03710-0)
Supplement: Supplementary file 1 — Supplementary Material 1 [file 394_2025_3710_MOESM1_ESM.docx]

| **Supplemental Table 1** |  |  | | | | | | | | | |
| --- | --- | --- | --- | --- | --- | --- | --- | --- | --- | --- | --- |
| **Metabolite** | **H chemical shift region (medium)** | **OPLS-EP 0h vs 3h Fatty fish** | | **OPLS-EP 0h vs 3h Soy protein** | | **OPLS-DA Δ3h**  **Red meat vs Fatty fish** | | **OPLS-EP AUC_min_  Red meat vs Fatty fish** | | **OPLS-DA AUC_min_ Red meat vs Fatty fish** | |
|  |  | *P* | increase/ decrease | *P* | increase/ decrease | *P* | **higher** | *P* | **higher** | *P* | **higher** |
| arginine + lysine^2^ | 1.85494 |  |  |  |  | 0.000040^1^ | *Fatty fish* | |  |  |  |
| arginine + unidentified^2^ | 1.61985 |  |  |  |  | 0.000040^1^ | *Fatty fish* | |  |  |  |
| creatine | 3.90292 | 0.000018^1^ | ↑ |  |  | 0.011867 | *Fatty fish* | 0.000119^1^ | *Fatty fish* | 0.000119 | *Fatty fish* |
| creatinine + myo-inositol + choline + cystine^2^ | 4.04441 |  |  | 0.000024^1^ | ↑ |  |  |  |  |  |  |
| cystine + unidentified^2^ | 4.0655 |  |  | 0.000018^1^ | ↑ |  |  |  |  |  |  |
| isoleucine | 0.99169 |  |  |  |  | 0.000040^1^ | *Fatty fish* | |  | 0.001932 | *Fatty fish* |
| leucine | 0.94776 |  |  |  |  | 0.000040^1^ | *Fatty fish* | |  |  |  |
| lipid | 5.18813 |  |  | 0.000021^1^ | ↑ |  |  |  |  |  |  |
| lipid | 5.29554 |  |  | 0.000018^1^ | ↑ |  |  |  |  |  |  |
| lipid | 5.31556 |  |  | 0.000018^1^ | ↑ | 0.000040^1^ | *Fatty fish* | 0.000046^1^ | *Fatty fish* | 0.000046^1^ | *Fatty fish* |
| lipid | 5.33558 |  |  | 0.000018^1^ | ↑ |  |  | 0.000378^1^ | *Fatty fish* | 0.000378^1^ | *Fatty fish* |
| lipid | 1.35176 |  |  | 0.000018^1^ | ↑ |  |  |  |  |  |  |
| lipid | 2.7534 |  |  | 0.000039 | ↑ | 0.018585 | *Red meat* | 0.000040^1^ | *Red meat* | 0.000040^1^ | *Red meat* |
| lipid | 2.77342 |  |  |  |  | 0.000040^1^ | *Fatty fish* | 0.000040^1^ | *Fatty fish* | 0.000040^1^ | *Fatty fish* |
| lipid + proline^2^ | 2.00346 |  |  | 0.000030 | ↑ |  |  |  |  |  |  |
| lipid | 2.2293 |  |  | 0.000018^1^ | ↑ |  |  |  |  |  |  |
| lipid + acetone^2^ | 2.20286 |  |  | 0.000018^1^ | ↑ |  |  |  |  |  |  |
| lipid | 1.57241 |  |  | 0.000018^1^ | ↑ |  |  |  |  |  |  |
| lipid | 0.871818 |  |  | 0.000021^1^ | ↑ |  |  |  |  |  |  |
| lipid | 0.906351 |  |  |  |  | 0.000091^1^ | *Fatty fish* | |  |  |  |
| threonine + unidentified^2^ | 4.23473 |  |  | 0.000030^1^ | ↑ |  |  |  |  |  |  |
| tyrosine + cystine + unidentified^2^ | 3.15359 |  |  |  |  | 0.000040^1^ | *Fatty fish* | |  |  |  |
| OPLS-EP= orthogonal projections to latent structures with effect projection, OPLS-DA= orthogonal projections to latent structures with discriminant analysis, AUC_min_=Area under curve minimum. Chemical shift region for the peak used for Wilcoxon signed rank test. Presented metabolites fulfil the selection criteria VIP top 20 and a loading score w > ± 0.1.*P* for Wilcoxon signed rank test is presented for all discriminating metabolites. 1) Significant Wilcoxon signed rank test after Bonferroni correction (*P*<0.0005). 2) overlapping peaks | | | | | | | | | | | |
